# The metabolic signature associated with the Western dietary pattern: a cross-sectional study

**DOI:** 10.1186/1475-2891-12-158

**Published:** 2013-12-11

**Authors:** Annie Bouchard-Mercier, Iwona Rudkowska, Simone Lemieux, Patrick Couture, Marie-Claude Vohl

**Affiliations:** 1 Institute of Nutrition and Functional Foods (INAF), Laval University, 2440 Hochelaga Blvd., Quebec G1V 0A6, Canada; 2 Department of Food Science and Nutrition, Laval University, 2425 de l’Agriculture St, Quebec G1K 7P4, Canada; 3 Endocrinology and Nephrology, CHU de Québec Research Center, 2705 Laurier Blvd., Québec G1V 4G2, Canada

**Keywords:** Dietary pattern, Western dietary pattern, Prudent dietary pattern, Acylcarnitine, Amino acids, Branched-chain amino acids, Metabolites

## Abstract

**Background:**

Metabolic profiles have been shown to be associated to obesity status and insulin sensitivity. Dietary intakes influence metabolic pathways and therefore, different dietary patterns may relate to modifications in metabolic signatures. The objective was to verify associations between dietary patterns and metabolic profiles composed of amino acids (AAs) and acylcarnitines (ACs).

**Methods:**

210 participants were recruited in the greater Quebec City area between September 2009 and December 2011. Dietary patterns had been previously derived using principal component analysis (PCA). The Prudent dietary pattern was characterised by higher intakes of vegetables, fruits, whole grain products, non-hydrogenated fat and lower intakes of refined grain products, whereas the Western dietary pattern was associated with higher intakes of refined grain products, desserts, sweets and processed meats. Targeted metabolites were quantified in 37 participants with the Biocrates Absolute IDQ p150 (Biocrates Life Sciences AG, Austria) mass spectrometry method (including 14 amino acids and 41 acylcarnitines).

**Results:**

PCA analysis with metabolites including AAs and ACs revealed two main components explaining the most variance in overall data (13.8%). PC1 was composed mostly of medium- to long-chain ACs (C16:2, C14:2, C14:2-OH, C16, C14:1-OH, C14:1, C10:2, C5-DC/C6-OH, C12, C18:2, C10, C4:1-DC/C6, C8:1 and C2) whereas PC2 included certain AAs and short-chain ACs (xLeu, Met, Arg, Phe, Pro, Orn, His, C0, C3, C4 and C5). The Western dietary pattern correlated negatively with PC1 and positively with PC2 (r = −0.34, p = 0.05 and r = 0.38, p = 0.03, respectively), independently of age, sex and BMI.

**Conclusion:**

These findings suggest that the Western dietary pattern is associated with a specific metabolite signature characterized by increased levels of AAs including branched-chain AAs (BCAAs) and short-chain ACs.

**Trial registration:**

NCT01343342

## Background

Single nutrients or single food components have been frequently studied in order to achieve a better understanding of their impact on health and on the development of chronic diseases. Many studies have also observed the effects of global diets or dietary patterns such as the Mediterranean diet on chronic diseases. Studying the effects of dietary patterns takes into account the interactions between nutrients [1]. Dietary patterns derived by principal component analysis (PCA) depict a portrait of the foods that are mainly consumed together within a population [1]. Dietary patterns, such as the Prudent (or Healthy pattern) and the Western dietary patterns have been associated positively or inversely with cardiovascular disease risk factors and mortality, as well as with certain types of cancer such as colorectal cancer [2-4]. Dietary patterns have also been associated with type 2 diabetes or related metabolic parameters. Schulze *et al*. [5] observed an association between the risk of type 2 diabetes and a dietary pattern high in foods such as sugar-sweetened soft drinks, refined grains as well as processed meats and low vegetable intake. In addition, Heidemann *et al*. [6] reported a decreased risk of type 2 diabetes with a dietary pattern characterised by high intakes of fruits and low intakes of foods such as high-caloric soft drinks and meats. These results have been confirmed by Esposito *et al*. [7] in a recent systematic review which observed that dietary patterns characterized by high intakes of fruit and vegetables, whole grains, fish, and poultry, and low intakes of red meat, processed foods, sugar-sweetened beverages, and starchy foods were associated with a reduced risk and a later development of type 2 diabetes.

Obesity pandemic represents a major health burden. In Canada, 26% of the adults were considered obese according to their body mass index (BMI) and when considering waist circumference, 37% were abdominally obese [8]. Obesity is closely related to insulin resistance [9]. However, the link between these two conditions is not well understood. Studying metabolites may help in further understanding the effects of diets, drugs and diseases at the cellular level and enhance our comprehension of the development of complex diseases such as type 2 diabetes (or insulin resistance) [10]. A few studies have investigated metabolic signatures in relation to insulin sensitivity or obesity [11-14]. For example, Newgard *et al*. [12] have observed a metabolic signature including branched-chain amino acids (BCAA) and short-chain acylcarnitines (ACs) among obese and insulin resistant individuals. These observations have also been replicated in Chinese and Asian-Indian individuals where a metabolic signature characterised by higher concentrations of amino acids (AAs) such as leucine/isoleucine, phenylalanine, tyrosine and methionine was also associated with insulin resistance [13]. Other studies observed the effects of dietary variables on metabolic signatures including ACs. For example, the effects of a lactovegetarien diet versus an omnivorous diet, the intake of fruits and vegetables, coffee and garlic intakes and hypocaloric dieting were studied [15-17]. Dietary patterns derived from cluster analysis have also been associated with specific metabolites [18].

To our knowledge, the metabolic signatures associated with the Western and the Prudent dietary patterns have never been studied. Therefore, the objective of this study was to investigate the metabolic signatures, composed of AAs and ACs derived from PCA, associated with the Western and the Prudent dietary patterns in a sample of overweight men and women. Associations between the Western dietary pattern and principal components (PCs) were observed.

## Methods

### Subjects

A total of 254 subjects were recruited to participate in this clinical trial from the greater Quebec City metropolitan area between September 2009 and December 2011 through advertisements in local newspapers as well as by electronic messages sent to university students/employees. To be eligible, subjects had to be non-smokers and free of any thyroid or metabolic disorders requiring treatment such as diabetes, hypertension, severe dyslipidemia, and coronary heart disease. Participants had to be aged between 18 and 50 years with a BMI between 25 and 40 Kg/m^2^. The subjects who had taken n-3 polyunsaturated fatty acid supplements during the six months preceding the study were excluded. A total of 210 subjects completed the study protocol which is described elsewhere [19] and were included in this cross-sectional study. All participants gave written informed consent and the experimental protocol was approved by the ethics committees of Laval University Hospital Research Center and Laval University. This trial was registered at clinicaltrials.gov as NCT01343342.

### Dietary assessment and food grouping

Dietary assessment and food grouping has been previously described [20]. Briefly, dietary intake of the past month was determined by a 91-items validated food frequency questionnaire (FFQ) [21] based on food habits of Quebecers, administered by a registered dietitian (RD). All the information was compiled and similar food items from the FFQ were grouped, as previously described [22]. Three criteria were used to form these groups: first, the similarity of nutrient profiles, second, the culinary usage of different types of food (similar to groups used in a previous study [23]) and third, the consideration of groups utilized in other studies to maintain consistency [1]. Food items from only thirty-five food groups were consumed by the participants in the present study. From these thirty-five food groups, eight were not normally distributed even after logarithmic transformation and were excluded as well. Consequently, twenty-seven foods groups were used for PCA to generate dietary patterns as described previously [20]. Briefly, two main dietary patterns were derived from PCA analysis. The Prudent dietary pattern, characterized by higher intakes of vegetables, fruits, whole grain products, non-hydrogenated fat and lower intakes of refined grain products, and the Western dietary pattern, associated with higher intakes of refined grain products, desserts, sweets and processed meats [20].

### Metabolite profiling

The Biocrates Absolute IDQ p150 (Biocrates Life Sciences AG, Austria) mass spectrometry method was used to quantify 163 metabolites for the first 40 of the 254 participants. Three participants were excluded because of extreme values (standard out of range), resulting in 37 participants. For this study ACs and AAs were the main focus thus, 41 ACs (AC*x*:*y*, where *x* denotes the number of carbons in the side chain and *y* the number of double bonds) and 14 AAs (proteinogenic + ornithine) were studied. Assays used 10 μL of plasma from each subject. The metabolite profiling was carried out according to the manufacturer's instructions at CHENOMX (Edmonton, AL, Canada). For all analyzed metabolites the concentrations are reported in μM. Furthermore, metabolites with standard out of range and/or for which more than half of the values were below the limit of detection were excluded. Thus, 29 ACs and 13 AAs were included in the analyses.

### Statistical analyses

Variables which were not normally distributed were logarithmically transformed. The distribution of glutaconyl-L-carnitine (C5_1_DC) was still not normally distributed after logarithmic transformation and thus was excluded from further analyses. The FACTOR procedure from Statistical Analysis Software (SAS) using PCA method was used to derive PCs describing metabolite signatures. Newgard *et al*. [11] described two main PCs when studying ACs and AAs which explained most of the variance in their data. In the present study, in order to determine the number of factors to retain, components with eigenvalue > 1, values at Scree test, variance explained (%) and the interpretability were considered. It was noticed that PC1 and PC2 had eigenvalues much higher (~8 and ~6, respectively) than the other PCs (<~3). Thus, the NFACTORS statement was added in the proc FACTOR procedure in order to retain only 2 main PCs and explain a maximum of variance. Metabolites with absolute factor loadings ≥ 0.50 were regarded as significant contributors to the PC. Using the SCORE procedure of SAS, each participant was given a score for each PC. These scores are calculated from the sum of metabolic signature groups multiplied by their respective factor loading. These scores reflect the degree of each participant’s metabolic signature conforming to PC1 and PC2.

Pearson correlations were used to detect associations between the Prudent and the Western dietary pattern scores with PC1 and PC2 scores. To further understand the relationships with PC1 and PC2 scores and dietary variables, partial correlations were performed with individual food groups (only the food groups which contributed to Prudent and Western dietary patterns) and macronutrients (expressed as energy percentages) adjusted for age, sex, BMI and energy intakes (only for the food groups). To facilitate interpretation, Prudent and Western dietary pattern scores as well as with food groups and macronutrients intakes were divided according to tertiles and associations with PC1 and PC2 were tested using the General Linear Model procedure implemented in SAS. A p-value <0.05 was considered significant. All statistical analyses were performed using SAS statistical software version 9.3 (SAS Institute, Inc., Cary, NC, USA).

## Results

### Descriptive characteristics and dietary patterns

Descriptive characteristics of the study participants are presented in Table 1. As described previously, there were two dietary patterns derived in this cohort, the Prudent and the Western dietary patterns [20]. The Prudent dietary pattern was characterised by high intakes of vegetables, fruits, whole grain products, non-hydrogenated fats and inversely associated with refined grain products food group and the Western dietary pattern by high intakes of refined grain products, desserts, sweets and processed meats [20]. In Table 2, dietary intakes, AC and AA concentrations according to dietary pattern score (low ≤ 0 or high > 0) are shown respectively for the Prudent and the Western dietary patterns. Individuals with high scores for the Prudent dietary pattern had lower saturated fat intakes than individuals with low scores. The opposite was observed for the Western dietary pattern scores. Regarding the associations between dietary patterns and cardiovascular disease risk factors, only a trend was observed for lower fasting insulin levels with higher scores for the Prudent dietary pattern (r = −0.32, p = 0.07). A positive association between fasting glucose levels and the Western dietary pattern was observed (r = 0.38, p = 0.03).

**Table 1:** Descriptive characteristics of the study participants

**Variables**	**All participants (n = 37)**
**Age (years)**	34.59 ± 9.16
**Sex (men/women)**	16/21
**BMI (Kg/m**^**2**^**)**	29.69 ± 4.17
**Waist circumference (cm)**	93.46 ± 11.89
**Systolic blood pressure (mmHg)**	108.03 ± 8.67
**Diastolic blood pressure (mmHg)**	71.89 ± 8.57
**Fasting glucose (mmol/L)**	5.01 ± 0.74
**Fasting insulin (pmol/L)**	84.57 ± 34.78
**Total-C (mmol/L)**	5.29 ± 1.35
**LDL-C (mmol/L)**	3.18 ± 1.13
**HDL-C (mmol/L)**	1.46 ± 0.48
**Triglycerides (mmol/L)**	1.43 ± 0.93
**ApoB (g/L)**	0.96 ± 0.30

Means ± SD.

**Table 2:** Dietary intakes and plasma AC and AA according to dietary pattern score

	**Low Prudent (≤0) (n = 20)**	**High Prudent (>0) (n = 17)**	**P-value**^**1**^	**Low Western (≤0) (n = 16)**	**High Western (>0) (n = 21)**	**P-value**^**1**^
**Dietary intakes**						
Carbohydrate (%)	49.38 ± 5.99	50.48 ± 6.37	0.35	51.91 ± 7.21	48.35 ± 4.73	0.35
Protein (%)	17.14 ± 2.78	18.46 ± 2.26	0.20	18.20 ± 2.66	17.40 ± 2.58	0.43
Total fat (%)	32.57 ± 6.15	31.93 ± 4.19	0.56	30.52 ± 5.89	33.61 ± 4.45	0.29
Saturated fat (%)	11.41 ± 1.83	9.55 ± 2.11	**0.004**	9.11 ± 2.08	11.65 ± 1.47	**0.0006**
Monounsaturated fat (%)	13.24 ± 3.06	13.48 ± 2.04	0.92	12.83 ± 2.98	13.75 ± 2.28	0.81
Polyunsaturated fat (%)	5.30 ± 1.53	6.24 ± 1.30	0.07	5.97 ± 1.57	5.55 ± 1.44	0.17
Cholesterol (mg)	329.88 ± 167.61	357.86 ± 188.90	0.84	263.76 ± 167.15	402.92 ± 160.78	0.07
Total fiber (g)	19.38 ± 4.83	30.43 ± 7.03	**<0.0001**	25.71 ± 7.53	23.51 ± 8.54	0.31
**Acylcarnitines (ACs)**						
C0	25.16 ± 5.07	27.50 ± 7.09	0.24	23.78 ± 6.73	28.10 ± 4.97	0.24
C2	5.55 ± 1.36	5.20 ± 1.55	0.57	5.33 ± 1.48	5.44 ± 1.44	0.76
C3	0.36 ± 0.14	0.29 ± 0.10	**0.04**	0.30 ± 0.12	0.34 ± 0.13	0.95
C4	0.18 ± 0.06	0.16 ± 0.07	0.44	0.16 ± 0.08	0.18 ± 0.05	0.46
C4:1-DC/C6	0.05 ± 0.01	0.06 ± 0.02	0.08	0.05 ± 0.02	0.05 ± 0.02	0.39
C5	0.11 ± 0.04	0.11 ± 0.03	0.64	0.10 ± 0.03	0.12 ± 0.04	0.39
C5-DC/C6-OH	0.01 ± 0.00	0.02 ± 0.00	**0.01**	0.01 ± 0.01	0.01 ± 0.00	0.62
C8:1	0.14 ± 0.08	0.12 ± 0.06	0.63	0.12 ± 0.08	0.13 ± 0.07	0.83
C10	0.16 ± 0.07	0.22 ± 0.13	0.07	0.17 ± 0.09	0.20 ± 0.11	0.47
C10:2	0.02 ± 0.01	0.03 ± 0.01	0.11	0.02 ± 0.01	0.03 ± 0.01	0.12
C12	0.06 ± 0.03	0.08 ± 0.03	0.06	0.06 ± 0.03	0.08 ± 0.03	0.31
C14:1	0.17 ± 0.03	0.21 ± 0.09	**<0.05**	0.19 ± 0.08	0.19 ± 0.07	0.95
C14:1-OH	0.01 ± 0.00	0.01 ± 0.01	0.54	0.01 ± 0.01	0.01 ± 0.01	0.41
C14:2	0.03 ± 0.01	0.04 ± 0.02	0.02	0.03 ± 0.02	0.03 ± 0.02	0.89
C14:2-OH	0.01 ± 0.00	0.01 ± 0.01	0.19	0.01 ± 0.00	0.01 ± 0.00	0.63
C16	0.08 ± 0.02	0.08 ± 0.04	0.37	0.07 ± 0.03	0.08 ± 0.03	0.45
C16:2	0.01 ± 0.00	0.01 ± 0.01	0.19	0.01 ± 0.00	0.01 ± 0.01	0.65
C18:2	0.04 ± 0.01	0.05 ± 0.01	**0.002**	0.04 ± 0.01	0.04 ± 0.01	0.42
**Amino acids**						
xleucine	183.95 ± 46.81	161.29 ± 26.70	**0.03**	152.94 ± 21.48	189.24 ± 44.08	**0.03**
Methionine	28.37 ± 5.14	26.54 ± 4.32	0.05	24.25 ± 4.23	30.02 ± 3.60	**0.0004**
Arginine	99.91 ± 32.87	95.81 ± 23.14	0.64	93.54 ± 27.05	101.44 ± 29.76	0.79
Phenylalanine	48.18 ± 8.10	46.56 ± 4.91	0.31	43.86 ± 4.55	50.16 ± 7.02	**0.007**
Proline	167.32 ± 58.74	157.68 ± 39.76	0.47	154.47 ± 44.03	169.30 ± 55.06	0.89
Ornitine	49.06 ± 17.18	52.61 ± 15.80	0.48	45.44 ± 17.75	54.69 ± 14.51	0.33
Histidine	93.59 ± 20.35	93.79 ± 13.96	0.94	93.55 ± 16.26	93.78 ± 17.74	0.65

Means ± SD.

^1^P-values of the GLM models are adjusted for age, sex and BMI.

P-values in bold were considered significantly different.

### Principal component analysis of the metabolites

**Figure 1:**
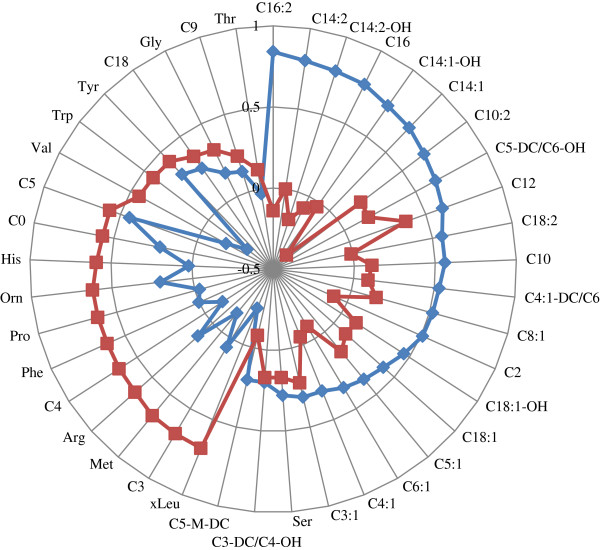
**ACs and AAs associated with PC1 and PC2.** Metabolites with absolute factor loadings ≥ 0.50 were regarded as significant contributors to the PC. The blue line and squares represent PC1 and the red line and squares represent PC2.

PC1 explained 7.97% of the variance in the data and PC2, 5.81%. As presented in Figure 1, PC1 was composed mostly of medium- to long-chain ACs (C16:2, C14:2, C14:2-OH, C16, C14:1-OH, C14:1, C10:2, C5-DC/C6-OH, C12, C18:2, C10, C4:1-DC/C6, C8:1 and C2) whereas PC2 was mainly composed of AAs and short-chain ACs (xLeu, Met, Arg, Phe, Pro, Orn, His, C0, C3, C4 and C5).

The Prudent dietary pattern was not correlated neither to PC1 nor to PC2 (r = −0.19, p = 0.26 and r = −0.21, p = 0.21, respectively). The Western dietary pattern, was correlated with PC2 (r = 0.34, p = 0.04). When further adjusted for the effects of age, sex and BMI, both PCs were associated with the Western dietary pattern. The Western dietary pattern correlated negatively with PC1 and positively with PC2 (r = −0.34, p = 0.05 and r = 0.38, p = 0.03, respectively). To further explore the association between the Western dietary pattern and PCs, subjects were divided into tertiles according to the Western dietary pattern score. The relationships between tertiles of the Western dietary pattern and PC1 and PC2 scores were not significant (p = 0.10 and p = 0.15, respectively).

**Figure 2:**
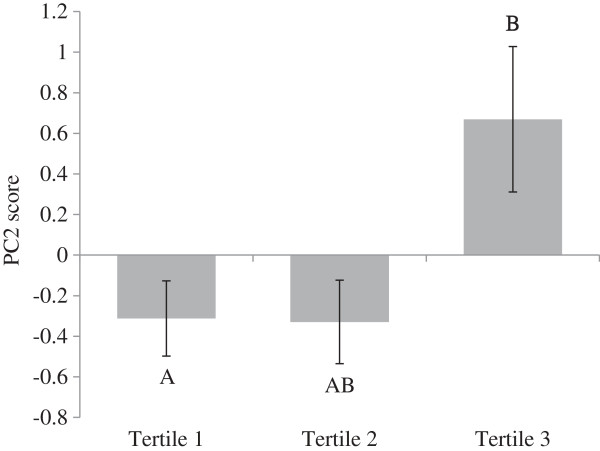
**PC2 scores according to tertiles of saturated fat intake.** PC2 scores and tertile of saturated fat intake (means ± SE). Means with different letters are significantly different. Means of saturated fat intake according to tertiles: tertile 1 (4.72-10.13%, n = 12), tertile 2 (10.29-11.30%, n = 13) and tertile 3 (11.51-14.72%, n = 12). Tertile 1 versus tertile 3: p = 0.005. Tertile 1 versus tertile 2: p = 0.40. Tertile 2 versus tertile 3: p = 0.05.

In order to achieve a better understanding of the impact of dietary variables on the metabolic signature, the possible correlations between each dietary variable (food groups contributing either to Prudent or Western dietary pattern and macronutrient intake (expressed in energy percentages)) and the scores for each PC were tested (adjusted for age, sex, BMI and energy intakes (only for food groups)), as presented in Table 3. Briefly, PC1 was not correlated with any food groups. PC2 was negatively correlated with fruit intake and positively associated with dessert intake (r = −0.38, p = 0.03 and r = 0.37, p = 0.04, respectively) adjusted for age, sex, BMI and energy intakes. For the macronutrients, expressed as energy percentages, a positive association for PC2 with total fat and saturated fat intakes was observed (r = 0.39, p = 0.02 and r = 0.50, p = 0.003, respectively). Interestingly, when dietary total fat, saturated fat, fruit and dessert were divided into tertiles, only saturated fat intake tertiles were different according to PC2 scores (Figure 2) (p = 0.01). As shown in Figure 2, saturated fat intakes ≤ 11.30% had negative PC2 scores, which indicate that their metabolic signature was not corresponding to PC2 characterised by higher concentrations of ACs and short-chain AAs. PC1 did not correlate with any macronutrients. In Table 4, partial correlations between dietary pattern scores and each AC and AA (only the metabolites which were associated with a PC) are shown. The Prudent dietary pattern score was positively associated with concentrations of C5-DC/C6-OH (glutaryl-L-carnitine) and C18:2 (octadecadienyl-L-carnitine). The Western dietary pattern score was positively associated with methionine and phenylalanine.

**Table 3:** Partial correlations between metabolite PCs and dietary pattern scores, food groups and macronutrient intakes

**Dietary variables**		**PC1**	**PC2**
**Dietary patterns**			
Prudent dietary pattern	r	−0.18	−0.25
	p^1^	0.31	0.15
Western dietary pattern	r	−0.34	0.38
	p^1^	**0.05**	**0.03**
**Food groups**			
Processed meats	r	−0.14	−0.12
	p^2^	0.45	0.52
Vegetables	r	−0.13	−0.27
	p^2^	0.46	0.13
Fruits	r	−0.10	−0.38
	p^2^	0.59	**0.03**
Whole grain products	r	−0.21	−0.24
	p^2^	0.23	0.18
Non-hydrogenated fats	r	−0.02	−0.17
	p^2^	0.93	0.34
Refined grain products	r	−0.14	0.04
	p^2^	0.43	0.85
Desserts	r	−0.09	0.37
	p^2^	0.60	**0.04**
Sweets	r	−0.18	0.25
	p^2^	0.32	0.16
**Macronutrient intakes**			
Total fat (%)	r	0.23	0.39
	p^1^	0.20	**0.02**
Saturated fat (%)	r	0.11	0.50
	p^1^	0.52	**0.003**
Monounsaturated fat (%)	r	0.28	0.28
	p^1^	0.10	0.10
Polyunsaturated fat (%)	r	0.06	0.07
	p^1^	0.74	0.70
Protein (%)	r	−0.00	−0.14
	p^1^	0.99	0.42
Carbohydrate (%)	r	−0.00	−0.29
	p^1^	0.99	0.10

^1^P-values are adjusted for age, sex and BMI.

^2^P-values are adjusted for age, sex, BMI and energy intakes.

P-values in bold were considered significantly different.

**Table 4:** Partial correlations between dietary pattern scores, ACs and AAs

**Acylcarnitines**	**Prudent dietary pattern**		**Western dietary pattern**	
C0	r	0.09	r	0.18
	p	0.60	p	0.32
C2	r	−0.05	r	0.07
	p	0.76	p	0.68
C3	r	−0.23	r	−0.02
	p	0.20	p	0.92
C4	r	−0.15	r	0.15
	p	0.41	p	0.41
C4:1-DC/C6	r	0.26	r	0.13
	p	0.14	p	0.46
C5	r	−0.26	r	−0.02
	p	0.14	p	0.91
C5-DC/C6-OH	r	0.35	r	−0.06
	p	**0.04**	p	0.74
C8:1	r	−0.20	r	−0.10
	p	0.25	p	0.56
C10	r	0.16	r	0.22
	p	0.36	p	0.20
C10:2	r	0.19	r	0.30
	p	0.29	p	0.08
C12	r	0.18	r	0.32
	p	0.32	p	0.07
C14:1	r	0.27	r	0.06
	p	0.12	p	0.73
C14:1-OH	r	0.07	r	0.11
	p	0.69	p	0.52
C14:2	r	0.28	r	0.08
	p	0.11	p	0.67
C14:2-OH	r	0.20	r	0.05
	p	0.27	p	0.78
C16	r	0.18	r	0.13
	p	0.30	p	0.47
C16:2	r	0.15	r	0.09
	p	0.39	p	0.62
C18:2	r	0.51	r	−0.17
	p	**0.002**	p	0.34
**Amino acids**				
xleucine	r	−0.33	r	0.30
	p	0.06	p	0.08
Methionine	r	−0.30	r	0.55
	p	0.08	p	**0.0008**
Arginine	r	−0.22	r	0.22
	p	0.21	p	0.21
Phenylalanine	r	−0.13	r	0.39
	p	0.46	p	**0.02**
Proline	r	−0.26	r	0.03
	p	0.14	p	0.87
Ornitine	r	0.16	r	−0.02
	p	0.37	p	0.92
Histidine	r	−0.25	r	−0.17
	p	0.16	p	0.34

P-values are all adjusted for age, sex and BMI.

P-values in bold were considered significantly different.

### Dietary intakes and individual metabolites

To further explore the impact of dietary intakes on metabolites, partial correlations were tested for each metabolite. Briefly, intakes of vegetables and fruits were positively associated with C18:2 (octadecadienyl-L-carnitine) (r = 0.49, p = 0.004, for both) and inversely associated with xleucine (r = −0.35, p = 0.05, for both). Fruit intakes were also inversely associated with methionine (r = −0.40, p = 0.02). The intakes of non-hydrogenated fats were positively associated with C14:1 (tetradecadienyl-L-carnitine) and C18:2 (octadecadienyl-L-carnitine) (r = 0.39, p = 0.02 and r = 0.46, p = 0.007, respectively) and inversely with histidine (r = −0.42, p = 0.01). The intakes of dessert were positively associated with three AAs, methionine, phenylalanine and xleucine (r = 0.49, p = 0.004, r = 0.40, p = 0.02 and r = 0.38, p = 0.03, respectively). The intake of sweets also was associated with methionine concentrations (r = 0.41, p = 0.02) and with C18:1-OH (hydroxyoctadecenoyl-L-carnitine) and C5:1-DC (glutaconyl-L-carnitine) (r = 0.37, p = 0.04 and r = 0.42, p = 0.01, respectively). When observing macronutrient intakes, saturated fat intakes expressed as energy percentages were positively associated with C5 (valeryl-L-carnitine) (r = 0.36, p = 0.04) and inversely with C18:2 (octadecadienyl-L-carnitine) (r = −0.46, p = 0.006). Monounsaturated fat intakes were positively associated with C8:1 (octenoyl-L-carnitine) (r = 0.50, p = 0.003) and inversely with C5-M-DC (methylglutaryl-L-carnitine) (r = −0.42, p = 0.01). Polyunsaturated fat intakes were also inversely associated with C5-M-DC (methylglutaryl-L-carnitine) (r = −0.35, p = 0.04) but as well with proline concentrations (r = −0.35, p = 0.04) and positively associated with C10:2 (decadienyl-L-carnitine) (r = 0.37, p = 0.03). Protein intakes expressed as energy percentages were associated with ornithine and histidine (r = 0.44, p = 0.009 and r = 0.42, p = 0.01, respectively). The opposite was observed for carbohydrate intakes which correlated inversely with ornithine concentrations (r = −0.55, p = 0.0008).

## Discussion

The Prudent and the Western dietary patterns from this study had many similarities with the dietary patterns described in the literature. The Prudent dietary pattern is usually associated with high consumption of vegetables, fruits and whole grain products whereas the Western dietary pattern relates to higher intakes of red and processed meats, refined grain products and sweets [24]. In this study, an association with higher fasting glucose was observed among individuals with high scores for the Western dietary pattern. Associations between dietary patterns and type 2 diabetes have been frequently observed and have been reviewed recently by Alhazmi *et al*. [25].

PC1 was composed mainly of medium- to long-chain ACs whereas PC2 was composed of short-chain ACs and AAs including the BCAA xleucine and the aromatic AA phenylalanine. Recent studies have shown a link between plasma levels of certain AAs and the risk of insulin resistance. Newgard *et al*. [12] have observed a metabolic signature among obese individuals characterised by a combination of BCAA, methionine, aromatic AAs and short-chain ACs (C3 and C5) which was related to insulin resistance. It has also been observed that increased levels of BCAA and aromatic AAs were associated with the risk of developing future type 2 diabetes [26]. Laferrère *et al*. [11] have studied the impact on ACs and AAs of weight-loss induced by gastric bypass surgery or by a hypocaloric diet. In their study, the first PC (mostly medium- to long-chain ACs) was associated with improved insulin sensitivity and the second PC (mostly AAs and short-chain ACs) was associated with an increase in insulin resistance. The gastric bypass surgery was associated with a decrease in short-chain ACs: C3, C4-DC and C5 ACs and AAs: alanine, leucine/isoleucine, phenylalanine and tyrosine. The ACs C3 and C5 have been demonstrated to be, at least partly, the products of AAs catabolism, especially BCAA (leucine/isoleucine and valine), possibly indicating an increase in enzymes related to BCAA catabolism [12]. In this study, no correlations were observed between PCs and fasting insulin or glucose levels. This could be due to the fact that the individuals from this cohort were healthy as well as only slightly overweight. Thus, their metabolic profile was not deteriorated leading to too subtle differences to be detected according to PCs scores.

Interestingly, scores for the Western dietary pattern were inversely associated with PC1 (medium- to long-chain ACs) and positively with PC2 (short-chain ACs and AAs including the BCAA xleucine). Xu *et al*. [15] compared the metabolite profile between a lactovegetarian diet with an omnivorous diet. Among the most different metabolites, there was glycine which was higher among lactovegeterians and phenylalanine which was lower among lactovegeterians compared to omnivorous controls. The authors hypothesised that phenylalanine concentrations may have been higher among the omnivorous group due to the intakes of animal proteins which contain more phenylalanine than proteins from vegetal sources. In our study, higher AAs levels in PC2 was not associated to an increase in total protein, animal protein or vegetal protein intakes (data not shown). In addition, protein intakes correlated positively with the Prudent dietary pattern and negatively with the Western dietary pattern. Thus, changes may be due to a modification in rates of protein turnover or AA catabolism. Differences in the expression of the enzyme responsible for BCAA catabolism (branched-chain α-ketoacid dehydrogenase (BCKD) complex) have been reported among obese rats compared to their lean counterparts [27]. BCKD’s activity was reduced among obese rats and also among diet induced obese mice, which were fed a diet containing from 45% to 60% energy from fat [27]. May *et al*. [16] have studied the effects on urine metabolomic profiles of a diet devoid of fruits and vegetables compared to a diet high in fruits and vegetables. They observed for the group deprived with fruits and vegetables, higher concentrations of short- to medium-chain ACs and higher concentrations of AAs and tricarboxylic cycle intermediates. They also hypothesised that these alterations could be due to a shift from glucose utilisation to fatty acid beta-oxidation. In our study, the Western dietary pattern was inversely associated with vegetable consumption (data not shown) and with a PC (PC2) characterised by higher concentrations of four short-chain ACs and seven AAs. In addition, an inverse association between fruit consumption and PC2 was observed in the present study. Thus, it seems that low fruit and vegetable intakes may be associated with a metabolic signature characterised by higher levels of shorter chain ACs and AAs.

When further examining the relationships between food groups and PCs, the most important correlation was observed between saturated fat intakes and PC2. Mechanisms behind these relations are unknown. Saturated fat intakes have been shown to be less potent activators than polyunsaturated fatty acids of an important transcription factor regulating fatty acid beta-oxidation, peroxisome proliferator-activated receptor alpha (*PPARA*) [28]. It has also been observed that oleate, compared to palmitate, the main saturated fat from the diet, increased mitochondrial fatty acid beta-oxidation [29]. However, other studies have reported the opposite. Stephenson *et al*. [30] have observed that among rats fed a «Western» diet (higher in fat, saturated fat and sucrose intakes) the activity of several mitochondrial enzymes involved in fatty acid beta-oxidation was increased. These discrepancies may be dependent of the overall effect of diet. Saturated fat alone may not have the same effect on fuel selection than when consumed in conjunction with higher intakes of sugary foods. In rat models, it has been observed that in long term, the rats fed a diet high in saturated fat and sucrose developed more severe symptoms of the metabolic syndrome than rats fed diets either high in saturated fat or high in sucrose alone [31].

Even though this cohort was generally healthy, differences in metabolic signatures have been observed and may be indicative of a higher or lower risk of future cardiometabolic diseases. A strength of this study is the analysis of dietary intakes grouped in dietary patterns from FFQ which represents real life intakes. Obviously, functional analyses are needed to understand underlying mechanisms behind these associations between dietary patterns and ACs and AAs concentrations in the plasma. One limitation of this study could come from the use of PCA. Results could be sample specific and strongly affected by subjective analytic decisions [1]. Nevertheless, to minimize subjectivity and allow data to be used in other studies, eigenvalue, Scree test and the literature were examined before selecting the number of PCs and for the dietary patterns, data from previous studies were also considered for food grouping.

## Conclusion

In conclusion, the results of the present study indicate a relationship between the Western dietary pattern and saturated fat intakes with a metabolic signature characterised by higher levels of short-chain ACs and AAs including BCAA and an aromatic AA. Individuals eating according to the Western dietary pattern or with high saturated fat intakes may increase their long term risk of cardiometabolic diseases possibly via small metabolic alterations.

## Abbreviations

AA: Amino acid; AC: Acylcarnitine; Arg: Arginine; BCAA: Branched-chain amino acid; BCKD: Branched-chain α-ketoacid dehydrogenase; BMI: Body mass index; FFQ: Food frequency questionnaire; His: Histidine; Met: Methionine; Orn: Ornitine; PC: Principal component; PCA: Principal component analysis; Phe: Phenylalanine; PPARA: Peroxisome proliferator-activated receptor; Pro: Proline; RD: Registered dietitian; SAS: Statistical analysis software; xLeu: xleucine.

## Competing interests

The authors declare that they have no competing interest.

## Authors’ contributions

IR, SL, PC, and MCV designed research; ABM conducted research with the research professionals; IR, SL, PC and MCV provided essential reagents or provided essential materials; ABM analyzed data and performed statistical analysis; ABM wrote paper; ABM, IR, SL, PC and MCV had primary responsibility for final content; All authors read and approved the final manuscript.
